# The Context Matters: Outcome Probability and Expectation Mismatch Modulate the Feedback Negativity When Self-Evaluation of Response Correctness Is Possible

**DOI:** 10.1155/2015/726798

**Published:** 2015-12-09

**Authors:** Anja Leue, Carmen Cano Rodilla, André Beauducel

**Affiliations:** ^1^Institute of Psychology, University of Kiel, 24118 Kiel, Germany; ^2^Institute of Psychology, University of Bonn, 53111 Bonn, Germany

## Abstract

Individuals typically evaluate whether their performance and the obtained feedback match. Previous research has shown that feedback negativity (FN) depends on outcome probability and feedback valence. It is, however, less clear to what extent previous effects of outcome probability on FN depend on self-evaluations of response correctness. Therefore, we investigated the effects of outcome probability on FN amplitude in a simple go/no-go task that allowed for the self-evaluation of response correctness. We also investigated effects of performance incompatibility and feedback valence. In a sample of *N* = 22 participants, outcome probability was manipulated by means of precues, feedback valence by means of monetary feedback, and performance incompatibility by means of feedback that induced a match versus mismatch with individuals' performance. We found that the 100% outcome probability condition induced a more negative FN following no-loss than the 50% outcome probability condition. The FN following loss was more negative in the 50% compared to the 100% outcome probability condition. Performance-incompatible loss resulted in a more negative FN than performance-compatible loss. Our results indicate that the self-evaluation of the correctness of responses should be taken into account when the effects of outcome probability and expectation mismatch on FN are investigated.

## 1. Introduction

Individuals often use external information in order to adapt their performance and their behavior in social and economic settings [1–3]. To this end, individuals typically evaluate whether their expectations, their performance, and the obtained feedback match. Some of these evaluation processes may occur relatively fast across time. That is why event-related potentials (ERP) of the electroencephalogram (EEG) are promising parameters, because these measurements provide important insights into fast changes of neural activity during feedback-related processes across time [4].

Miltner et al. found that feedback about the accuracy of performance elicits a negative deflection occurring about 250 ms postfeedback at frontocentral electrode sites [5]. This negative deflection has been originally named as error-related negativity to feedback (feedback-ERN or fERN) [5, 6] and later as feedback negativity (FN) because the FN is not restricted to error feedback. In tasks that provided positive feedback following correct responses and negative feedback following incorrect responses, a more positive FN amplitude has been observed for positive feedback, whereas the FN had a negative deflection following negative feedback [7–9]. It has, therefore, been proposed that the FN is driven by the positivity of outcome rather than by its negativity. According to Foti et al., the FN tracks the relative outcome valence in the context of the alternative outcomes, suggesting that FN depends on one's expectations immediately before the outcome [7].

In their influential work, Holroyd and Coles investigated the response-locked ERN as an internal signal of outcome expectation and the FN as an external signal of outcome expectation in a probabilistic learning task [6]. Their investigation of the ERN and FN within one task demonstrates that these ERPs are related to the same neurological systems. In their probabilistic learning task, six stimuli were associated with different outcome probability and feedback conditions. By performing the task, participants learned the association of stimulus, correct response, and feedback. When the association of stimulus, response, and feedback has been learned, the stimulus becomes a precue for the expected response-feedback association. Holroyd and Coles compared the response-ERN and the FN and demonstrated that in the 50% outcome probability condition the response-ERN was less pronounced (more positive) than the FN [6]. In the 50% condition, correct responses could be followed by reward but also by punishment. Thus, participants could not learn from the obtained feedback so that a self-evaluation of the correctness of their responses was impossible. Consequently, participants could not evaluate whether the feedback matched or mismatched their performance.

In contrast to the 50% outcome probability condition of the probability learning task [6], the correctness of responses could be learned in the 100% outcome probability condition, because the feedback was compatible with the responses. Accordingly, in the 100% outcome probability condition, the response-ERN was more pronounced than the FN. This finding suggests that the response served as an internal signal of outcome expectation when participants had learned the association between stimuli and required responses. The work of Holroyd and Coles [6] provided important insights into the relation between ERN and FN in a probabilistic learning task. Particularly, their model can explain the common neurological basis of these ERPs and the amplitude of ERN and FN. Both ERN and FN can represent a negative reinforcement learning signal that is conveyed to the functioning of the ACC. Nevertheless, several conditions determining FN remain to be investigated in order to improve our understanding of the neural mechanisms of FN.

Knowledge of the correctness of a response was not a priori given in Holroyd and Coles [6] and could be learned in the 100% outcome probability condition, but it could not be learned in the 50% outcome probability condition. Accordingly, low outcome probability was associated with unknown correctness of responses, and high outcome probability was associated with knowledge of the correctness of response. Therefore, the effects of outcome probability and the effect of self-evaluation of the correct responses on the FN could not be disentangled in Holroyd and Coles [6]. It is therefore possible that the effects attributed to outcome probability were not effects of the outcome probability alone but effects of different degrees of knowledge concerning the (self-evaluated) correctness of the responses. In consequence, it is impossible to infer from Holroyd and Coles [6] whether outcome probability (without any effect of outcome probability on knowledge of result) has an effect on FN.

Moreover, Hajcak et al. [10] applied three different precues in order to manipulate the predictability of outcome (named as outcome probability in Holroyd and Coles [6]) in a gambling task. The numbers 1, 2, or 3 were presented on a screen to inform participants how many doors would be associated with a win feedback. Based on this information, participants were asked to guess whether they would win or lose money. Because the number of doors and the doors that were associated with monetary win or loss changed from trial to trial, participants could not have any knowledge on the correctness of their responses and they could not learn an association of correctness of responses and feedback. This might be the reason why precues in Hajcak et al. [10] did not become a salient stimulus of outcome probability so that no significant FN differences were observed between predictable and less predictable outcome.

Briefly, when outcome probability varies in a context that allows for a self-evaluation of correctness of one's performance, outcome probability may affect feedback processing and FN. One reason for an effect of outcome probability on FN could be that it provides a frame of reference for the evaluation of outcome valence. For example, when two outcomes with a different valence follow a correct response and when the outcome probability is at chance level, individuals will not know whether a more negative outcome or a more positive outcome will follow their correct response. Since two outcomes can follow the correct response when outcome probability is at chance level, the more positive outcome is likely to be evaluated against the more negative outcome. In contrast, when the outcome following a correct response is completely predictable (i.e., 100% outcome probability), only one outcome is possible following correct responses. When no alternative outcomes are possible, no comparative outcome evaluation is possible. Therefore, an outcome probability at chance level may induce another relative outcome valence for correct responses compared to a 100% outcome probability, which implies a complete certitude of the outcome. Such differences of relative outcome valence induced by different outcome probability can occur even when the absolute valence of the outcome is the same in the two outcome probability conditions. However, the certitude that no other outcome is possible in the 100% condition can only occur, when individuals have perfect knowledge of the correctness of response related to a given outcome. Therefore, we aimed at investigating the effect of outcome probability on FN under a condition of constant knowledge of result. One method to investigate the effect of outcome expectations on the FN amplitude is to define outcome probability by means of precues in a simple task that allows for knowledge of the correctness of response.

To sum up, we expect an effect of outcome probability on feedback valence. The FN has been initially suggested to represent an emotional evaluation of feedback valence on a good-bad dimension with a more negative FN amplitude following negative compared to positive feedback [10–12]. Holroyd et al. have demonstrated that feedback valence varies depending on context and that outcome is evaluated in relation to other possible outcomes in a specific context ([13]; see also [14–16]). Accordingly, the aim of our study was to investigate whether the context of possible outcomes is affected by outcome probability when knowledge of correct responses is given.

Beyond outcome probability and feedback valence, we aim at introducing a second concept during feedback processing that we name performance-compatibility. To be more concrete, when the correct response is a priori known and the task is pretty simple, individuals have an internal representation of the correctness of their responses and might establish an expectation of the feedback valence that should derive from their performance. According to the first-indicator hypothesis [17], which has been derived from the reinforcement-learning-theory (RLT [6]) to predict variations of feedback processing depending on an individual's performance, performance monitoring might induce an “internal signal” of outcome expectation. In simple tasks with predefined correct and incorrect responses that are known to the participants, they are usually able to evaluate whether they responded correctly or incorrectly. When an internal performance-based outcome expectation corresponds to the received feedback, the FN should not indicate an expectation mismatch. In contrast, when individuals responded correctly and obtained negative feedback, internal outcome expectation and received feedback do not match. Similarly, a mismatch between an internal performance-based signal of outcome expectation and external feedback might occur after responding incorrectly and receiving a feedback that does not correspond to the erroneous response (i.e., no-loss or even win). In these examples, external feedback is incompatible with an individual's internal signal of outcome expectation that is due to performance monitoring in a simple task. Thus, when performance tasks allow for a self-evaluation of the correctness of the responses, self-evaluation of performance might serve as an internal signal of outcome expectation. In the following, an external feedback that is compatible with the performance-based signal of outcome expectation will be named “performance-compatible feedback,” whereas external feedback that is incompatible with the performance-based outcome expectation will be named “performance-incompatible feedback.”

In sum, we aimed at investigating effects of outcome probability (2 levels: 100% versus 50% condition) and feedback valence (2 levels: no-loss versus loss) on the FN. Outcome probability and feedback valence were independently manipulated in a task condition with constant a priori knowledge of the correct response and performance-compatible outcome. More specifically, we expect outcome probability at chance level to establish a more complex context for the evaluation of feedback valence than 100% outcome probability. The 100% outcome probability condition will result in the complete certitude of the resulting outcome/feedback when knowledge of result is available. Accordingly, the relative valence of a no-loss feedback following correct responses will be considered being less negative in the 50% condition when loss feedback is also possible following correct responses compared to the 100% condition, when only no-loss feedback is possible for correct responses. Therefore, the FN is expected to be less pronounced (less negative) for no-loss following correct responses in the 50% condition than for no-loss following correct responses in the 100% condition (hypothesis a). In contrast, incorrect responses are followed by loss in the 100% condition and can be followed by no-loss or loss in the 50% condition. Accordingly, the relative valence of loss should be more negative in the 50% condition (when no-loss may also occur following incorrect responses) than in the 100% condition where no-loss is the only outcome to be expected (hypothesis b). Moreover, we aimed at investigating effects of expectation mismatch on the FN when performance-compatibility (2 levels: performance-compatible versus incompatible outcome) and feedback valence (2 levels: no-loss versus loss) were independently manipulated in a task condition with a priori knowledge of the correct response and an outcome probability at chance level (50%). In the 50% outcome probability condition, performance-compatible no-loss and performance-compatible loss were thought not to induce an expectation mismatch. However, performance-incompatible no-loss should induce a better-than-expected mismatch and performance-incompatible loss should evoke a worse-than-expected mismatch. Accordingly, we expected performance-incompatible feedback to result in a more negative FN than performance-compatible feedback (hypothesis c).

## 2. Materials and Method

### 2.1. Sample

A total of *N* = 22 students (*n* = 8 male) of the University of Bonn, Germany, participated voluntarily in this study (age: M = 21.82 years, SD = 2.46, and range: 19–28 years). Participants of this study were selected from a larger project if they had at least 20 artefact-free EEG epochs per task condition (cf. EEG recording and quantification). At the beginning of this study, we obtained a written informed consent from all participants according to the Declaration of Helsinki. The ethical board of the German Foundation of Psychologists provided a positive evaluation of the experimental protocol of the present study. All participants were right-handed according to the handedness inventory of Oldfield [18] and had normal or corrected-to-normal vision.

### 2.2. Precue Go/No-Go Task

All participants performed a go/no-go task comprising a total of 512 trials. Reinforcement-related versions of this go/no-go task were previously tested [19, 20]. However, the precued version of the present go/no-go task has been newly developed. Go and no-go stimuli were presented with equal frequency (i.e., 256 go stimuli and 256 no-go stimuli). Go and no-go stimuli were white colored geometric forms consisting of a square and a circle, respectively. In the present go/no-go task we used two feedback precues representing outcome probability. One precue (indexed by “#”) signaled an outcome probability of one (100% condition). That is, participants knew that correct responses to go and no-go stimuli always resulted in a no-loss feedback (i.e., 0 Cents). In contrast, when participants responded too slowly to go stimuli (500 to 1,000 ms poststimulus) or incorrectly to no-go stimuli, they always received a monetary loss feedback (i.e., −2 Cents or −4 Cents; Table 1). Feedback was always performance-compatible in the 100% outcome probability condition. For ethical reasons and in order to avoid irritating the participants, no performance-incompatible feedback was introduced in the 100% outcome probability condition (Table 1). Providing performance-incompatible feedback in the 100% outcome probability condition would have meant that participants obtain monetary loss in each case of correct responses and no-loss in each case of incorrect responses (cf. Table 1). In consequence, participants would have often been punished although they responded in accordance with the instruction. In order to avoid these problems, performance-incompatible feedback was exclusively provided in trials with an outcome probability at chance level (50% condition indexed by “?” as a precue).

In trials with an outcome probability at chance level, 128 trials were associated with loss in case of incorrect responses or no-loss following correct responses (performance-compatible feedback). The other 128 trials were associated with loss in case of correct responses and with no-loss in case of incorrect responses (performance-incompatible feedback). Altogether, there were 256 trials presenting feedback precues of high outcome probability and 256 trials with an outcome probability at chance level. Trials with a 50% versus 100% outcome probability were presented in a pseudorandom order to minimize effects of precue feedback anticipation. Table 1 gives an overview of the outcome probability precues, the response types, and the feedback types.

**Figure 1:**
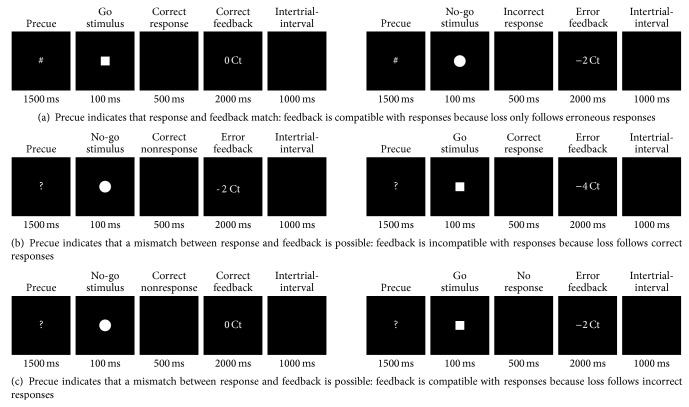
Examples of trial sequences (we do not present examples of all possible combinations of outcome probability precue, performance, and feedback valence).

The timing of a trial sequence in the 50% and in the 100% outcome probability conditions was identical. The precue was presented for 1,500 ms followed by a go stimulus or a no-go stimulus lasting 100 ms. Responses to go stimuli were required within 500 ms, whereas participants were asked to withhold responses to no-go stimuli. Feedback was presented for 2,000 ms. The intertrial-interval was 1,000 ms (Figure 1).

### 2.3. Measures

After finishing the go/no-go task, participants evaluated the unpleasantness of the no-loss feedback and the monetary loss feedback in the 50% and in the 100% outcome probability trials on a 9-point Likert scale (ranging from 1 = less unpleasant to 9 = highly unpleasant). Regarding trials with a high outcome probability precue (“#”), participants were asked to evaluate the unpleasantness of no-loss feedback following correct responses and the monetary loss feedback following too slow responses to go stimuli or following erroneous responses to no-go stimuli. For trials with an outcome probability precue at chance level (“?”), participants rated the unpleasantness of no-loss and monetary loss feedback.

### 2.4. Procedure

Participants were recruited through announcements on a bulletin board, flyer, and an electronic platform announcing recent research projects at the University of Bonn, Germany. Participants who were interested in this study were instructed in a telephone call to omit alcohol use, to avoid unusual caffeine and nicotine consumption, and to avoid taking other stimulating substances the day before EEG recording. All participants reported that they had never had a neurological or a mental disorder. The room where the EEG was recorded was sound-attenuated and well-lit. Presentation V12.1 (Neurobehavioral Systems, Albany, NY) was used to present the go/no-go task on a 20-inch flat screen. Participants sat in a comfortable chair (about 95 cm from the screen) while performing the task. Participants were instructed to respond very fast to go stimuli and to withhold responses to no-go stimuli. They were informed about the two different outcome probability precues and about the different types of feedback. They were told that no-loss feedback would follow correct responses and loss feedback would follow incorrect responses in trials with a high outcome probability precue. Participants were also informed that in trials with an outcome probability precue at chance level correct responses could be associated with no-loss (performance-compatible) but also with loss (performance-incompatible worse-than-expected outcome) and that incorrect responses could be followed by no-loss (performance-incompatible better-than-expected outcome) or by monetary loss (performance-compatible outcome). Participants were given a starting budget of 7.70 € for each of the two task blocks and they were asked to lose as few money as possible. In trials with an outcome probability at chance level, all participants lost money after correct responses in trials with a performance-incompatible feedback and after incorrect responses in trials with performance-compatible feedback (Table 1). In trials with an outcome probability of 100%, participants lost money exclusively following incorrect responses. All participants performed 12 practice trials with each trial type occurring at least once (Table 1). Subsequently, the EEG was recorded during the 50-minute lasting task. Participants were given a 2-minute break after 25 minutes (for statistical analysis FN epochs were collapsed across both task blocks). The experimenter sat in an adjacent room, where EEG data and behavioral data were saved to disk. Each examination including preparation of EEG recording, feedback ratings, experimental task, and debriefing took about 90 minutes. Participants received a basic payment of 15 €. The mean amount of additional payment was M = 10.84 € (SD = 0.65; range: 8.70 €–11.50 €).

### 2.5. EEG Recording and Quantification

**Figure 2:**
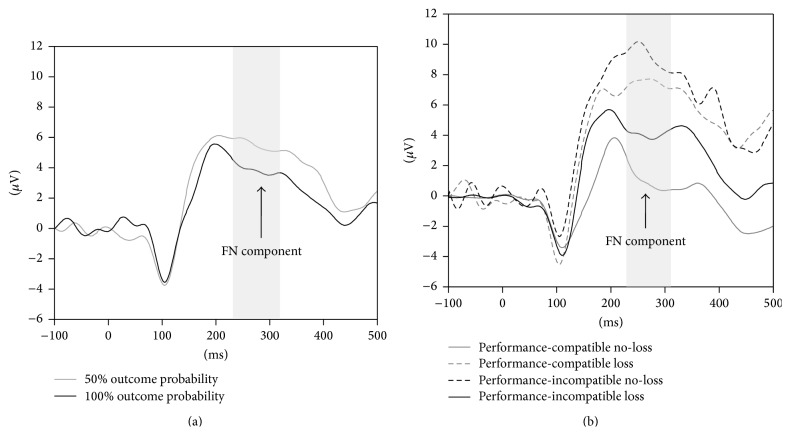
(a) Feedback-locked grand averages at Fz for trial with 50% versus 100% outcome probability and (b) feedback-locked grand averages at Fz in trials with performance-compatible and performance-incompatible no-loss versus loss (50% outcome probability condition).

**Figure 3:**
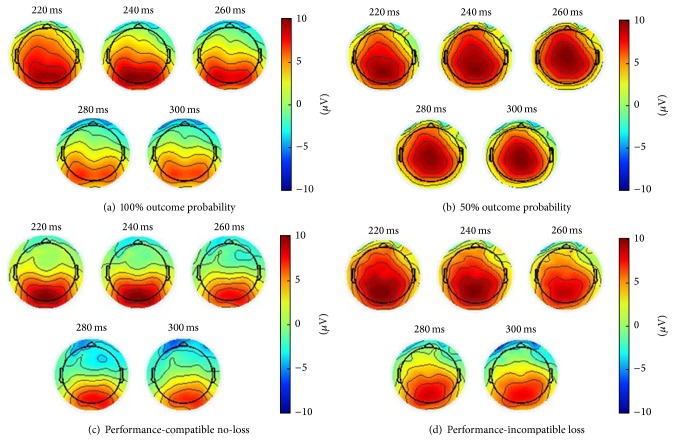
Topographical maps (a) of the 100% outcome probability condition and (b) of the 50% outcome probability condition. Topographical maps (c) of the 50% outcome probability condition with performance-compatible no-loss and (d) of the 50% outcome probability condition with performance-incompatible loss.

EEG recording, quantification, and analysis were conducted in accordance with the guidelines for the study of human ERPs [21]. The EEG was recorded using the Active Two software (Biosemi, Amsterdam, Netherlands) with 64 active scalp electrodes based on the extended 10/20 system [22]. The electrooculogram was recorded from two horizontal electrodes placed beyond the epicanthi of both eyes and from one vertical electrode placed about 1 cm below the right eye. Ground electrodes during data acquisition were the Common Mode Sense (CMS) active electrode and the Driven Right Leg (DRL) passive electrode. The impedances of all electrodes were below 25 kΩ. Data were recorded without any online reference. Offline EEG analysis was performed using EEGLab v12.0.2.0b based on MATLAB 7.14.0.739. The EEG was sampled at 512 Hz. The feedback-locked data were high-pass filtered with 0.1 Hz and low-pass filtered with 20 Hz [23] and referenced to linked mastoids (P9 and P10 of the Biosemi headcap). An automated infomax decomposition algorithm (ICA) was applied to correct for ocular artifacts. Further technical and muscle artifacts were rejected when the EEG signal exceeded ±85 *μ*V. Data were baseline corrected using the average activity in the interval between 100 and 0 ms before feedback presentation and subtracting this average activity from the subsequent data points. As becomes apparent from the grand averages (Figure 2), the FN component occurred between 220 ms and 310 ms postfeedback and was quantified as the most negative voltage in the before-mentioned time window for the different feedback types (i.e., baseline-to-peak FN amplitude). Topographical maps have been performed for the two levels of outcome probability in trials with performance-compatible feedback and for performance-compatible no-loss as well as performance-incompatible loss in the 50% outcome probability condition (Figures 3(a) and 3(b)).

For statistical analysis, at least 20 FN epochs were available for each participant and each task condition (i.e., for 100% versus 50% outcome probability, for no-loss versus loss in trials with performance-compatible feedback, for performance-compatible versus performance-incompatible feedback, and for no-loss versus loss feedback in the 50% outcome probability FN epochs). The available number of 20 FN epochs per condition suggests that the FN was reliably measured (cf. [24]).

### 2.6. Statistical Analysis

We conducted repeated measures ANOVAs for feedback ratings, behavioral data, and the FN amplitude. Gender was inserted as a between-subjects factor in all repeated measures ANOVAs. Although we did not conceptually focus on gender differences in this study, we performed the ANOVAs with gender as well as without gender as a between-subjects factor because gender has sometimes been found to modulate the FN amplitude [10, 25]. The repeated measures ANOVA for feedback ratings included outcome probability (100% outcome probability signaled by “#” versus 50% outcome probability signaled by “?”) and feedback valence (no-loss versus monetary loss) as repeated measures factors, because participants evaluated the received feedback types on these dimensions.

For response times we conducted a repeated measures ANOVA with outcome probability and response type (correct responses to go stimuli associated with no-loss feedback versus incorrect responses associated with no-go stimuli with monetary loss; because at the time of the responses the feedback has not yet been provided, we named this repeated measures factor “response type” instead of “feedback valence” in contrast to analyses of the FN) as repeated measures factors. Another repeated measures ANOVA was conducted for the percentage of correct responses to further analyze response accuracy. To parallel the analysis of response accuracy with the analysis of response times, outcome probability and response type (correct responses to go stimuli versus correct nonresponses to no-go stimuli) were again applied as repeated measures factors in the ANOVA for the percentage of correct responses.

For the FN amplitude, we conducted the following repeated measures ANOVAs: the first ANOVA included exclusively the FN amplitudes in performance-compatible trials. We analyzed the topographical effect of the FN amplitude in an ANOVA containing position (Fz, FCz, Cz, CPz, and Pz; cf. [26]), outcome probability (“#” versus “?”), and feedback valence (no-loss versus loss) as repeated measures factors (ANOVA I, cf. Table 1). In a second ANOVA including exclusively FN epochs with a 50% outcome probability, position (Fz, FCz, Cz, CPz, and Pz; cf. [26]), performance-compatibility (performance-compatible versus performance-incompatible), and feedback valence (no-loss versus loss) were inserted as repeated measures factors (ANOVA II, cf. Table 1). Robustness of the results was tested for this electrode position where the FN amplitude was most pronounced. Effect sizes are reported in terms of partial eta square (*η*_*p*_^2^).

## 3. Results

### 3.1. Feedback Ratings

Participants rated the unpleasantness of no-loss (0 Cents) and monetary loss (collapsed across −2 Cents and −4 Cents) in the 50% and in the 100% outcome probability condition. The outcome probability main effect of the unpleasantness rating showed a tendency, *F*(1,20) = 3.62,  *p* = 0.07,  *η*_*p*_^2^ = 0.15. Unpleasantness ratings for the 100% outcome probability condition (“#”; M = 3.20, SE = 0.32) were slightly higher than the unpleasantness ratings for the outcome probability precue at chance level (“?”; M = 2.72, SE = 0.23). The feedback valence main effect was significant, *F*(1,20) = 33.49, *p* < 0.01, *η*_*p*_^2^ = 0.63, suggesting that monetary loss (M = 4.53, SE = 0.47) was more unpleasant than no-loss (M = 1.39, SE = 0.23). The outcome probability × feedback valence interaction of the unpleasantness rating showed a tendency, *F*(1,20) = 3.11, *p* = 0.09, *η*_*p*_^2^ = 0.13. The gender main effect was not significant for the unpleasantness rating, *F*(1,20) < 1, ns. The same was true for the outcome probability × gender interaction, *F*(1,20) < 1, ns, and for the feedback valence × gender interaction, *F*(1,20) = 3.07, *p* = 0.10, *η*_*p*_^2^ = 0.10. Unpleasantness ratings of no-loss and loss feedback did not significantly correlate with the baseline-to-peak FN amplitude at Fz (for further FN analyses see Section 3.3) for the corresponding task conditions, *r*s(22) ≤ 0.21, ns.

### 3.2. Behavioral Data

For response times, the outcome probability main effect was not significant, *F*(1,20) = 1.06, *p* = 0.32 (Table 2). The response type main effect of response times, *F*(1,20) = 29.64, *p* < 0.01, *η*_*p*_^2^ = 0.60, suggested that response times of correct responses to go stimuli were significantly longer than response times of erroneous responses to no-go stimuli (Table 2). The outcome probability × response type interaction of response times was also significant, *F*(1,20) = 9.49, *p* < 0.01, *η*_*p*_^2^ = 0.32. This interaction indicated that the difference of response times for correct response times and error response times was significantly larger in trials with an outcome probability at chance level (M_correct−incorrect_ = 158.68 ms, SE_correct−incorrect_ = 29.89) compared to trials of the 100% outcome probability condition (M_correct−incorrect_ = 84.59 ms, SE_correct−incorrect_ = 19.57). The gender main effect, the outcome probability × gender interaction, and the feedback valence × gender interaction for response times were all nonsignificant, *F*s(1,20) < 1, ns.

Repeated measures ANOVA for the percentage of correct responses revealed a significant main effect of outcome probability, *F*(1,20) = 16.70, *p* < 0.01, *η*_*p*_^2^ = 0.46. The percentage of correct responses was higher in the 100% outcome probability condition (M = 93.64, SE = 1.32) compared to the 50% outcome probability condition (M = 82.48, SE = 2.74). Moreover, the response type main effect was significant for the percentage of correct responses, *F*(1,20) = 5.77, *p* < 0.05, *η*_*p*_^2^ = 0.24. The percentage of correct responses to no-go stimuli (M = 91.15, SE = 2.01) was higher than the percentage of correct responses to go stimuli (M = 84.50, SE = 2.49). This indicates that participants rather produced errors of omission to go stimuli than commission errors to no-go stimuli. The interaction of outcome probability × response type for the percentage of correct responses, *F*(1,20) = 9.52, *p* < 0.01, *η*_*p*_^2^ = 0.32, suggested that the percentage of correct responses was higher for go stimuli in the 100% outcome probability condition (M = 93.33, SE = 2.39) compared to the 50% outcome probability condition (M = 73.64, SE = 4.01), *F*(1,20) = 6.37, *p* < 0.01, *η*_*p*_^2^ = 0.57, but not for no-go stimuli, *F*(1,20) = 2.52, *p* = 0.13 (100% outcome probability: M = 93.95, SE = 0.92; 50% outcome probability: M = 88.35, SE = 3.67). The gender main effect, the outcome probability × gender interaction, and the response type × gender interaction for the percentage of correct responses were not significant, *F*s(1,20) < 1, ns. Results for behavioral data were not substantially altered when gender was not included as a between-subjects factor.

### 3.3. FN Amplitude

**Figure 4:**
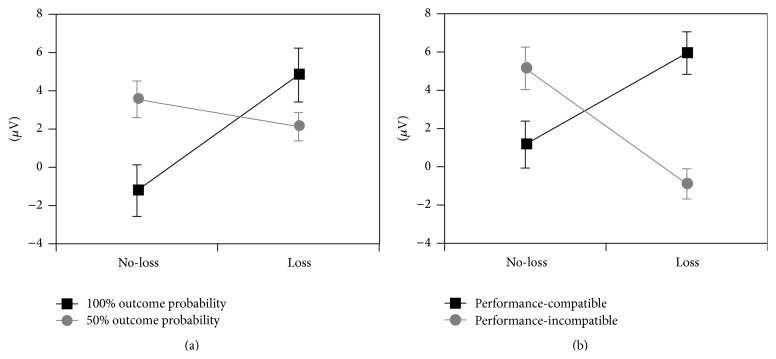
Variations of the FN amplitudes (a) depending on outcome probability and feedback valence (including exclusively performance-compatible trials) and (b) depending on performance-compatibility and feedback valence (including exclusively trials with 50% outcome probability).

In ANOVA I (including exclusively performance-compatible FN epochs), the position main effect was significant, *F*(4,80) = 10.10, *p* < 0.01, Greenhouse-Geisser *ε* = 0.35, *η*_*p*_^2^ = 0.34. Deviation contrasts revealed that the FN amplitude was most negative at Fz (M = −7.77 *μ*V, SE = 0.75) compared to the average of the other electrodes (M_averaged_ = −6.16 *μ*V, SE_averaged_ = 0.48), *F*(1,20) = 8.77, *p* < 0.01, *η*_*p*_^2^ = 0.31. Subsequently, we first report the analyses for Fz, FCz, Cz, CPz, and Pz followed by the analyses that were exclusively conducted at Fz. The outcome probability main effect was not significant, *F*(1,20) < 1, ns. The feedback valence main effect was significant, *F*(1,20) = 5.58, *p* < 0.05, *η*_*p*_^2^ = 0.22. In contrast to prior FN findings for feedback valence, the FN amplitude was more pronounced (i.e., less positive) following no-loss (M = 1.16 *μ*V, SE = 0.94) compared to loss (M = 3.47 *μ*V, SE = 0.93) when feedback was always compatible with knowledge of results. The outcome probability × feedback valence interaction was significant, *F*(1,20) = 20.89, *p* < 0.01, *η*_*p*_^2^ = 0.51. This interaction could be traced back to the following main effects: in performance-compatible trials that were associated with no-loss, the outcome probability main effect was significant, *F*(1,20) = 11.90, *p* < 0.01, *η*_*p*_^2^ = 0.37. As expected, the no-loss FN amplitude was less negative in the 50% outcome probability condition compared to the 100% outcome probability condition (hypothesis a, Figure 4(a)). The outcome probability main effect in performance-compatible loss trials, *F*(1,20) = 4.44, *p* < 0.05, *η*_*p*_^2^ = 0.18, indicated that the FN amplitude was more pronounced (i.e., less positive) in trials with 50% outcome probability compared to trials with 100% outcome probability (hypothesis b). All these findings in trials with performance-compatible feedback were also significant at Fz (i.e., the electrode position where the FN amplitude was most pronounced). The gender main effect was not significant, *F*(1,20) < 1, ns, and results were not substantially altered when gender was not included as between-subjects factor.

In ANOVA II (including exclusively FN epochs with a 50% outcome probability), the position main effect was also significant, *F*(4,80) = 17.27, *p* < 0.01, Greenhouse-Geisser *ε* = 0.46, *η*_*p*_^2^ = 0.46. As in ANOVA I, deviation contrasts suggested that the FN amplitude was more pronounced (i.e., less positive) at Fz (M = 0.44 *μ*V, SE = 0.80) compared to the other electrodes (M_averaged_ = 3.43 *μ*V, SE_averaged_ = 0.82), *F*(1,20) = 42.56, *p* < 0.01, *η*_*p*_^2^ = 0.68. Accordingly, results were first reported for Fz, FCz, Cz, CPz, and Pz and subsequently at Fz. The performance-compatibility main effect was not significant, *F*(1,20) = 1.08, *p* = 0.31, *η*_*p*_^2^ = 0.05. The feedback valence main effect showed a tendency, *F*(1,20) = 2.95, *p* = 0.10, *η*_*p*_^2^ = 0.13. However, the performance-compatibility × feedback valence interaction was significant, *F*(1,20) = 23.14, *p* < 0.01, *η*_*p*_^2^ = 0.54. Following no-loss, the performance-compatibility main effect was significant, *F*(1,20) = 12.97, *p* < 0.01, *η*_*p*_^2^ = 0.39, indicating that the FN amplitude was more pronounced (i.e., less positive) following performance-compatible feedback compared to performance-incompatible feedback. Following loss feedback, a significant performance-compatibility main effect was also observed, *F*(1,20) = 24.28, *p* < 0.01, *η*_*p*_^2^ = 0.55, with a more negative FN amplitude following performance-incompatible feedback (i.e., correct responses) compared to performance-compatible feedback (i.e., incorrect responses, hypothesis c). The gender main effect was not significant, *F*(1, 20) < 1, ns. All these results were robust when analyzed at Fz except the feedback valence main effect in performance-compatible trails which was no longer significant.

## 4. Discussion

The present study separately manipulated effects of outcome probability and performance-based outcome expectation on the FN amplitude in a go/no-go task. Moreover, feedback valence was manipulated in conjunction with outcome probability and in conjunction with performance-compatibility. Outcome probability was manipulated by means of precues (indicating an outcome probability of 100% or 50%). Moreover, the go/no-go task was very simple and participants were able to self-evaluate the correctness of their responses. Therefore, participants could have an outcome expectation based on the self-evaluation of response correctness combined with the outcome probability that was indicated by precues. We investigated the compatibility of the received feedback with the outcome expectation based on the self-evaluation of response correctness (i.e., performance-compatibility). The main results for outcome probability can be summarized as follows: as expected, the FN amplitude after correct responses was less negative for no-loss in the 50% condition than for no-loss in the 100% condition (hypothesis a). The FN following loss in performance-compatible trials was more negative in the 50% condition than in the 100% condition (hypothesis b). Since outcome probability modulated the FN and the relative feedback valence, the result on absolute feedback valence was in contrast to conventional findings on feedback valence showing a more negative FN amplitude following negative compared to positive feedback. This underlines the impact of outcome probability on feedback valence.

The main results for performance-compatibility are as follows: following loss feedback, the FN amplitude was more pronounced when feedback was performance-incompatible (i.e., participants had responded correctly but were punished) compared to performance-compatible feedback (i.e., participants received monetary loss following incorrect responses) (hypothesis c). Moreover, in trials with performance-incompatible feedback, unjustified monetary loss (i.e., participants received monetary loss despite correct responses) resulted in a more pronounced FN amplitude than unjustified no-loss (i.e., participants did not lose any money despite erroneous responses). These findings provide evidence that expectation mismatch occurred when we manipulated performance-compatibility of feedback and feedback valence, and they suggest a relation of FN with (un)fairness evaluations (see further discussion below). Although knowledge of result was continuously available, outcome probability altered the FN magnitude. Whereas Holroyd and Coles [6] demonstrated an effect of outcome probability on FN that was related to different degrees of knowledge of result, we showed an effect of outcome probability on FN even when knowledge of result was continuously available. This indicates that outcome probability may alter the context in that the relative valence of feedback is evaluated. The relative valence of no-loss following correct responses was more positive in the 50% outcome probability condition than in the 100% condition because loss could also follow correct responses in the 50% condition. Conversely, the relative valence of loss following incorrect responses was more negative in the 50% outcome probability condition than in the 100% condition because no-loss could also occur following incorrect responses in the 50% condition. Thus, the alterations of the feedback context resulting from different outcome probabilities extend the results of Holroyd et al. [13]. The finding illustrating that outcome probability shapes the context in that feedback valence is evaluated also suggests that the relative or anticipated valence of feedback can be more important for individuals than the absolute valence of feedback.

Regarding expectation mismatch, our data provide new evidence that performance-incompatible loss feedback evoked a more intense expectation mismatch than performance-compatible loss feedback in trials with an outcome probability at chance level and with knowledge about the correct response. Individuals used their internal performance-related signal in order to establish an outcome expectation and the FN indicates whether outcome was contrary to correct performance (i.e., loss following correct responses). We conclude from our results that the evaluation of outcome in relation to outcome probability and performance overruled direct evaluations of feedback valence. Individuals did not only evaluate whether outcome was negative or positive, but they integrated expectations that were formed based on outcome probability precues, their performance, and the obtained outcome.

When negative feedback is given following correct performance, more socially related concepts of fairness versus unfairness are likely to be activated. At least since Greenberg, the relation between performance and the corresponding evaluation has been a basis for fairness and justice models [27]. Our data highlight the importance of this relation, since the FN was more negative when monetary loss occurred following correct responses compared to loss following incorrect responses. Performing correctly and being punished is a classical situation that should evoke feelings of unfairness. Thus, unfairness evaluations appear to result from the discrepancy between internal (performance-related) and external (valence-related) signals evoking expectation mismatch. Our results suggest that theories on fairness [2] should integrate the interplay of internal and external outcome signals. A differentiation of determinants of fairness concerns (e.g., internal performance evaluation, external feedback valence) would expand existing research on human moral cognition [3] and would improve our understanding of fairness processing. In contrast, unfairness concerns are less likely to occur when individuals do not have a clear representation of their performance, for example, due to the fact that response intervals are so short that they cannot evaluate whether responses result in a positive outcome or response errors have been committed so marginally beyond a response limit [17]. Stahl [17] has demonstrated that response time errors (evoking error-related negativity, ERN) that were slightly beyond a response time limit resulted in a more pronounced FN amplitude but did not affect the response-locked ERN. Consequently, when responses do not provide a clear internal signal of outcome expectation, an external feedback signal as represented by the FN amplitude is necessary for outcome evaluation. In our study, we did not focus on the differentiation of marginal and far beyond response times as an internal feedback signal by means of ERN. However, implications of the first-indicator hypothesis [6, 17] are of interest for the interpretation of our findings. When individuals have a clear internal performance-based feedback signal because the task is pretty simple–as in our study–but feedback does not correspond to the self-evaluated performance, the FN reflects an expectation mismatch that is likely to comprise unfairness concerns. To this end, the interplay of self-evaluated correct performance and feedback valence in situations that induce ambiguous outcome expectations should be investigated in unfairness research. In order to further our understanding of internal signals of outcome expectation, it could also be promising to experimentally manipulate self-generated expectations versus cue-related expectations [28].

## 5. Conclusion

Our data illustrate that precue-related outcome probability establishes a context of feedback and thereby affects the processing of feedback valence. No-loss feedback following correct responses resulted in a less negative FN for outcome probability at chance level than for 100% outcome probability. Thus, outcome probability has an effect of FN amplitudes even when knowledge of result is available throughout all trials. Moreover, in a condition with an outcome probability at chance level performance-compatibility modulated FN. In ambiguous outcome situations (i.e., outcome probability at chance level), performance-incompatible feedback that followed correct responses resulted in a more negative FN than performance-compatible feedback. Situations that signal the probability of less predictable or unpredictable outcome and situations that incorporate outcome valence that is incompatible with individuals' performance evaluation are likely prerequisites of unfairness concerns.

## Figures and Tables

**Table 1:** Overview of outcome probability precues, response types, and feedback valence. The mean number of artefact-free FN epochs is given in the note.

Outcome probability precue	Response	Feedback valence	
100% outcome probability precue “#”	Correct	No-loss	—
	Incorrect	—	Monetary loss

50% outcome probability precue “?”	Correct	No-loss	Monetary loss
	Incorrect	No-loss	Monetary loss

*Note.* To have at least five artefact-free FN epochs for monetary loss trials, FN epochs with a monetary loss of 2 Cents and 4 Cents were collapsed. Thus, variations of the FN amplitude depending on magnitude of monetary loss were not investigated in this study.

**Table 2:** Means and standard errors (in parentheses) of response times (in ms).

Outcome probability precue	Response	Feedback valence	
		No-loss	Monetary loss
100% outcome probability precue “#”	Correct	244.75 (5.65)	—
	Incorrect	—	160.17 (19.36)

50% outcome probability precue “?”	Correct	267.74 (8.55)	262.95 (45.12)
	Incorrect	117.47 (127.22)	86.45 (31.81)
